# Subducting serpentinites release reduced, not oxidized, aqueous fluids

**DOI:** 10.1038/s41598-019-55944-8

**Published:** 2019-12-20

**Authors:** F. Piccoli, J. Hermann, T. Pettke, J. A. D. Connolly, E. D. Kempf, J. F. Vieira Duarte

**Affiliations:** 1 University of Bern, Institute of Geological Sciences, Balzerstrasse 1+3, 3012 Bern, Switzerland; 2 Department of Earth Science, Swiss Federal Institute of Technology, Zurich, Switzerland

**Keywords:** Mineralogy, Petrology

## Abstract

The observation that primitive arc magmas are more oxidized than mid-ocean-ridge basalts has led to the paradigm that slab-derived fluids carry SO_2_ and CO_2_ that metasomatize and oxidize the sub-arc mantle wedge. We combine petrography and thermodynamic modelling to quantify the oxygen fugacity (*f*O_2_) and speciation of the fluids generated by serpentinite dehydration during subduction. Silicate-magnetite assemblages maintain *f*O_2_ conditions similar to the quartz-fayalite-magnetite (QFM) buffer at fore-arc conditions. Sulphides are stable under such conditions and aqueous fluids contain minor S. At sub-arc depth, dehydration occurs under more reducing conditions producing aqueous fluids carrying H_2_S. This finding brings into question current models in which serpentinite-derived fluids are the cause of oxidized arc magmatism and has major implications for the global volatile cycle, as well as for redox processes controlling subduction zone geodynamics.

## Introduction

**Figure 1:**
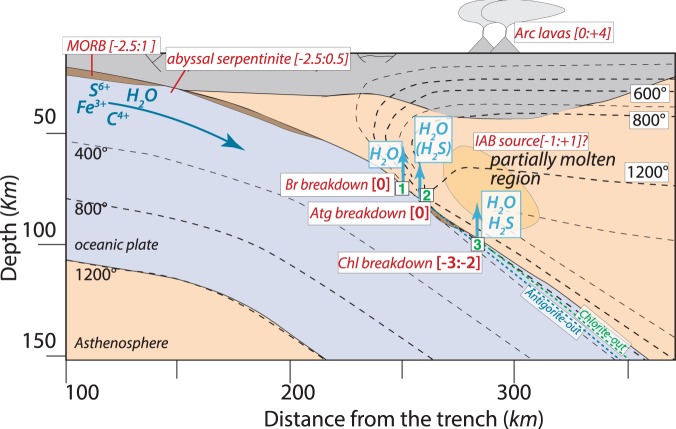
Cartoon of a subduction zone illustrating the subduction of hydrated, relatively oxidized, oceanic lithosphere and where fluids releasing reactions from ultramafic rocks occur at the slab surface, along with calculated fluid *f*O_2_ expressed as ∆logQFM (in square bracket) and composition. Thermal model for Central Honshu from Syracuse *et al*.^58^ and temperatures are given in degrees Celsius. MORB and arc lava oxygen fugacity values are from Christie *et al*.^59^, Bézos and Humler^60^, Lee *et al*.^40^; abyssal serpentinite from Deschamps *et al*.^61^; island arc basalt (IAB) source from Ballhaus^37^ and Parkinson and Arculus^39^.

Extensive studies on arc lavas have found that arc basalts are more oxidized than mid-ocean ridge basalts^1–3^. Most of these works discount magmatic low-pressure differentiation as an oxidation mechanism and attribute the oxidized nature of arc lavas to the oxidation of the mantle wedge magma source by slab-derived fluids. Intense hydrothermal interaction of oceanic lithosphere with seawater results in the precipitation of carbonates, sulphates and ferric iron oxides^4–6^ producing a km-wide zone on top of subducted slabs that is comparatively oxidized with respect to the mantle wedge (Fig. 1). Hydrothermally altered oceanic lithosphere contains oxidized species (Fe^3+^, C^4+^, S^6+^) and these elements are present in different concentrations in sediments, mafic crust and mantle lithologies that are introduced into subduction zones^7–9^. The potential release of such oxidized species during dehydration reactions may be coupled with a change in the rock redox state and thus explain the oxidation of the mantle wedge by infiltrating fluids^10^.

Hydrous ultramafic rocks (i.e. serpentinites) are generally considered as the main water carriers and a major source of fluids in subducting oceanic lithosphere at fore-arc to subarc depth^11^. Mantle peridotites, exposed to the seafloor on slow/ultraslow spreading ridges during tectonic extension, are serpentinized and represent a sink for water, and redox sensitive elements such as carbon and sulphur^6,12^ (Fig. 1). In particular sea-floor oxidation leads to the precipitation of abundant magnetite and serpentine containing ferric iron^12–16^. Consequently, serpentinites have been regarded by several researchers as principle carriers of excess oxygen into subduction zones^9,17–19^, with oxidised sulphur released in the fluid phase as the main means of transport of redox budget from the slab to the locus of partial melting. This has led to the current paradigm that oceanic serpentinization and oxidation control the redox potential of fluids released by dehydration^9,20^.

We investigated the silicate-oxide-sulphide relationships in subducted serpentinites that document the effects of the three key dehydration reactions that occur between fore-arc and sub-arc conditions. This information is combined with thermodynamic modelling along a prograde *P-T* path in order to constrain the evolution of rock-buffered *f*O_2_ during these dehydration reactions. Our findings show that in the presence of magnetite, silica activity imposed by the different silicate mineral assemblages controls oxygen fugacity in hydrous peridotitic systems. We argue that in hydrated ultramafic rocks dehydration reactions take place at rock-buffered conditions and that this buffering has important consequences for the solubility and speciation of sulphur. Thermodynamic calculations show that sulphur occurs predominantly as reduced rather than oxidized fluid species when liberated from hydrous mantle rocks at sub-arc depth and thus serpentinite-derived fluids are unlikely to be responsible for the oxidized character of subduction zone magmas, contrary to claims in previous works.

## Silicate-Oxide-Sulphide Relations in High-Pressure Serpentinites, Chlorite-Harzburgites, and Garnet Metaperidotite

**Figure 2:**
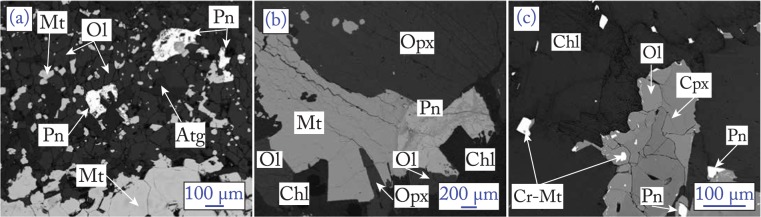
Backscatter images of hydrated ultramafic rocks from Zermatt (**a**), Cerro del Almirez (**b**) and Cima di Gagnone (**c**) showing equilibrium texture between silicates, magnetite and pentlandite. Ol = olivine; Atg = antigorite; Pn = pentlandite; Mt = magnetite; Cr-Mt = chromium-magnetite; Opx = orthopyroxene; Chl = chlorite; Cpx = clinopyroxene.

The first major dehydration reaction in subducted antigorite serpentinites is

1 $${\rm{Brucite}}+{\rm{Antigorite}}={\rm{Olivine}}+{\rm{Chlorite}}+{\rm{Fluid}}$$

and occurs at 500–550 °C and fore-arc depth for typical slab geotherms, liberating up to ~2.2 wt.% H_2_O^21^. This reaction is well-documented in the Zermatt-Saas unit, North-Western Alps, Switzerland, which experienced peak metamorphic conditions of 550 °C, 2.5 GPa^22,23^. The newly formed olivine is in textural equilibrium with antigorite, chlorite, magnetite^23,24^ and Fe-Ni sulphides (pentlandite, ±pyrrhotite) (Fig. 2a).

The antigorite breakdown reaction

2 $${\rm{Antigorite}}={\rm{Olivine}}+{\rm{Orthopyroxene}}+{\rm{Chlorite}}+{\rm{Fluid}}$$

is the most important dehydration reaction in serpentinites and occurs at 650–700 °C and 1.6–1.8 GPa, liberating between 5–12 wt.% H_2_O^11^. This reaction is well recorded at Cerro del Almirez, Spain^25,26^ where the reaction products olivine, orthopyroxene and chlorite (chlorite-harzburgite hereafter) coexist with magnetite and pentlandite (±pyrrhotite; ±ilmenite). Previous studies have suggested that hematite is also stable in the chlorite-harzburgite^27^. However, our examination revealed that hematite occurs only along cracks in association with retrograde talc and chrysotile/lizardite mixtures and as exsolution lamellae from high-*T* ilmenite. These textures indicate that hematite is most likely a product of retrogression and does not constrain prograde *f*O_2_ conditions.

The final dehydration reaction liberates 2.5–3 wt.% H_2_O and is related to the consumption of chlorite at 750–800 °C and 2.8 GPa at subarc depth by the reaction:

3 $${\rm{Chlorite}}+{\rm{Orthopyroxene}}={\rm{Garnet}}+{\rm{Olivine}}+{\rm{Fluid}}$$

This reaction is documented in lenses of chlorite-harzburgite and garnet metaperidotite at Cima di Gagnone, Central Alps, Switzerland^28,29^. The peak paragenesis of the chlorite-harzburgite comprises olivine, orthopyroxene, clinopyroxene, chlorite, with accessory Cr-rich magnetite, pentlandite and pyrrhotite (this study^30,31^,). In garnet peridotite Cr-Al-spinel, Cu-sulphide, Fe-Ni-Cu and Fe-Cu sulphide, pentlandite, Fe-Ni arsenide, and ilmenite are in equilibrium with the peak silicate assemblage composed of olivine, orthopyroxene, clinopyroxene and garnet (this study^30,31^,). The presence of arsenides and di-sulphides such as chalcopyrite in garnet peridotite indicates higher S fugacity conditions compared to chlorite-harzburgite^32^.

## Calculated Oxygen Fugacities

**Figure 3:**
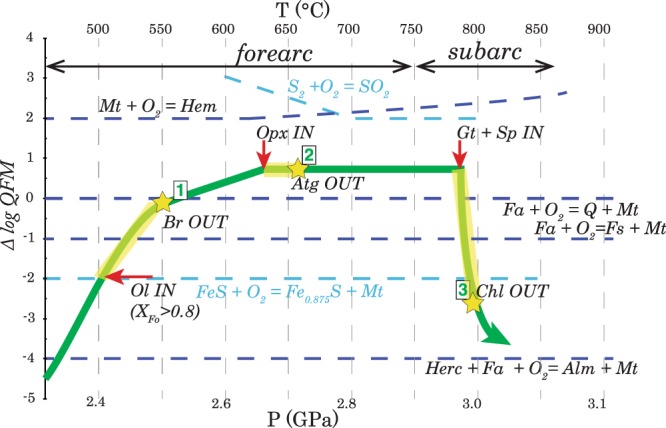
*Pressure-Temperature*-*f*O_2_ evolution of hydrated ultramafic rocks. Dehydration reactions occur along a divariant field (yellow highlighted areas). Stars indicate the end of the stability field of hydrated phases: brucite, antigorite and chlorite. On the y-axis, *f*O_2_ is expressed as ∆logQFM to eliminate the *P-T* dependence of the *f*O_2_ absolute value (see also Fig. S1). Ol = olivine; Br = brucite; Opx = orthopyroxene; Atg = antigorite; Gt = garnet; Sp = spinel; Chl = chlorite; Hem = hematite; Mt = magnetite; Fa = fayalite; Q = quartz; Fs = ferrosilite; Herc = hercynite; Alm = almandine; FeS = troilite.

The equilibrium of Fe-bearing silicates with magnetite reflects the *f*O_2_ in metaperidotite^33^. Iron is the most abundant redox sensitive element in the subducted serpentinites. Previous studies have focused on calculating the redox budget (amount of moles of electrons that need to be added to the rock to reach the reference state^34^) of the silicate assemblage of serpentinites^32^ and on the antigorite breakdown reaction (2)^20^. However, redox conditions prevailing upon reaction (1) (Br-out) and reaction (3) (Chl-out) have remained largely unconstrained. In this study we investigated a suite of samples of progressively dehydrating ultramafic rocks and show how the silicate-oxide assemblages can be used to reconstruct the entire prograde *P-T-f*O_2_ evolution. We modelled conditions from 450 to 850 °C and from 2 to 3 GPa along a linear subduction geotherm of 15 °C/km (details and modelling parameters in Supplementary Information, Fig. S1). The results are reported with reference to the commonly used oxygen fugacity buffer quartz-fayalite-magnetite (QFM) in delta log notation to eliminate the *P-T* dependence of the absolute oxygen fugacity value (Fig. 3). The QFM equilibrium is given by:

4 $${\rm{Fayalite}}+{{\rm{O}}}_{2}={\rm{Magnetite}}+{\rm{Quartz}}$$

Metamorphic olivine is present from the onset of reaction (1) and the fayalite component is decreasing with increasing temperature (Fig. S2). We have shown that magnetite is the stable oxide upon dehydration reaction (1) and (2). Free quartz is not present but a(SiO_2_) is buffered by coexisting silicates such as brucite and antigorite or olivine and orthopyroxene. This means that knowing the *P-T* conditions for reaction (1) and (2), and with magnetite being present, we can constrain the oxygen fugacity by modelling the change in Si activity (i.e. change in silicate assemblage). Olivine-free antigorite serpentinite from Zermatt-Saas has the lowest Si activity, with the Si buffering assemblage being brucite + antigorite (~log(aSiO_2_) = −2.3). Such a low Si activity indicates that these samples are the most reduced (4 log unit below the QFM; Fig. 3). With increasing *T* and olivine crystallization, the fayalite component in olivine decreases and Si activity increases up to ~log(aSiO_2_) > −2 (Fig. S2) driving the *f*O_2_ to higher values, close to QFM. This corresponds to an increase in *f*O_2_ by 4 log units (Fig. 3).

With continued subduction, antigorite breaks down by a continuous reaction to form orthopyroxene (reaction 2) and Si-activity is buffered by orthopyroxene + olivine (Fig. S3). Samples from Cerro del Almirez show that dehydrating antigorite serpentinites have a redox solid buffer (magnetite + olivine + orthopyroxene). The fayalite-ferrosilite-magnetite buffer is one log unit below QFM. However, because of the high Mg# of olivine and orthopyroxene (i.e., molar Mg/(Fe + Mg) ∼ 0.90, measured by electron microprobe), *f*O_2_ is ~0.5 log units higher than the QFM buffer (Figs. 3 and S1). Consequently, the antigorite-out reaction (2) does not cause an increase in *f*O_2_. It is worth noting that antigorite dehydration at lower pressure, such as in Cerro del Almirez (1.6–1.8 GPa), will occur at identical *f*O_2_ conditions because the Si activity is still buffered by olivine + orthopyroxene, with the only difference being that the peak assemblage includes tremolite^26,35^.

The last dehydration reaction occurs at higher *P-T* conditions (3 GPa − 770 °C), where chlorite + orthopyroxene react to produce garnet + olivine (reaction 3, Fig. 2c). The oxide assemblage at *HP-HT* conditions includes magnetite together with spinel. Magnetite + spinel in equilibrium with olivine + garnet is a solid buffer and indicates that reaction (3) occurs at buffered oxygen fugacity conditions. As the reaction proceeds magnetite is progressively replaced by Cr-Al bearing spinel with low ferric iron content (this study^30^,). This depletion in ferric iron requires that during chlorite dehydration, oxygen fugacity decreases by 2–3 log units below the QFM (Fig. 3). At the end of the reaction, Fe^3+^ is mainly hosted in clinopyroxene and garnet^36^. This implies that at higher temperature, when magnetite is completely consumed, redox conditions in the residual anhydrous metaperidotite are governed by equilibria involving Fe^3+^ bearing silicates^36,37^ and thus will be sensitive to the bulk rock Fe^3+^/Fe^tot^.

## Fluid Speciation

**Figure 4:**
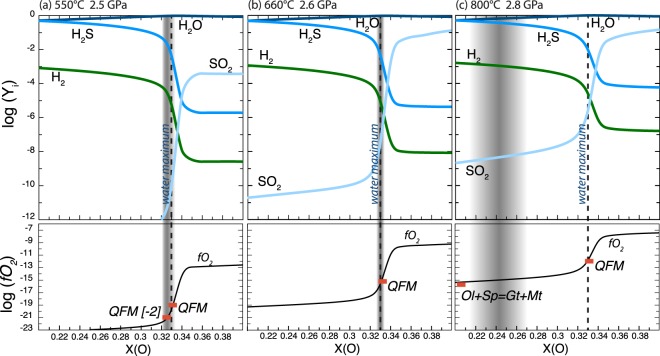
Isobaric-isothermal diagrams for H-O-S fluid in equilibrium with sulphide (pyrrhotite buffer) illustrating the mole fraction (Y_i_) of fluid species as a function of X(O) (oxygen molar ratio: O/(O + H) in the fluid phase). The oxygen fugacity (black line) and the position of mineral buffers (red marks) are also reported (lower boxes). The dashed line indicates the water maximum (X(O) = 0.33) and the grey field indicates the rock buffered oxygen fugacity.

As the *P-T*-*f*O_2_ conditions for each dehydration reaction have been established, we can estimate the H-O-S molecular speciation of the fluid. Here we provide the predicted speciation of a sulphur bearing fluid liberated from serpentinite and chlorite-harzburgite. At the conditions prevailing at the brucite and antigorite-out reactions (*f*O_2_ increasing from −2 to 0 ΔQFM and QFM buffered, respectively; Fig. 4a,b) a HOS-fluid will be situated close to the water maximum (oxygen molar ratio XO = 1/3) with very minor H_2_S, and H_2_. In both cases, total sulphur mobilized by the fluid is extremely low (Fig. 4a,b). This result is also supported by the petrographic observations on samples from Zermatt and Cerro del Almirez, where the stability of Fe-Ni sulphide across dehydration reactions (1) and (2) documents subordinate mobilization of sulphur.

Reaction 3 occurs close to the spinel + olivine = garnet + magnetite buffer (Fig. 3). Our calculations show that at these *P-T*- *f*O_2_ conditions fluids are reduced. Sulphide dissolution in such a reduced environment will produce H_2_S bearing fluids (Fig. 4c). It is noteworthy that a comparatively large amount of S is released at these *P-T-f*O_2_ conditions (Fig. 4c). This result is consistent with the de-sulphidation observed in garnet peridotite (i.e., presence of Fe-Ni arsenides replacing pentlandite).

Absolute S solubility in high-pressure fluids are strongly dependent on the chosen models: molecular species or electrolytes (Table S1). Here we report the results for molecular species fluids (see also Fig. S4). These values are to be considered as conservative estimates. We modelled sulphur solubility at rock buffered conditions using S bulk content ranging from 0.05 to 0.2 wt.%. These values are in the range of reported values for abyssal serpentinites (ranging from 320 to 2300 ppm)^6^. Both the obtained phase diagrams predict the stability of magnetite with pyrrhotite and/or pyrite, thus indicating that the initial sulphur content does not affect the silicate-magnetite solid buffer, in agreement with the large *f*O_2_ stability field predicted for the assemblage magnetite + pentlandite by Evans *et al*.^32^. In such a case, solubility does not depend on bulk concentration. Our model predicts that H_2_S in fluid at brucite-out and antigorite-out conditions does not exceed tens of ppm (20 and 70 ppm, respectively). At chlorite-out condition, S solubility increase by one order of magnitude and H_2_S content reaches hundreds of ppm (300 ppm). Importantly, both models (molecular species and electrolytes) indicate that S solubility in fluids equilibrated with ultramafic rocks increases prominently from fore-arc to subarc conditions.

## Implications for Redox Processes and The Global S Cycle

It is widely accepted that the sub-arc mantle is oxidized^19,38,39^. This has led to the paradigm that oxidation of the mantle wedge is related to the oxidizing properties of slab derived fluids^1,2,19,40^. We have demonstrated that in subducting ultramafic rocks magnetite-silicate equilibria buffer the *f*O_2_ and that during the major dehydration reactions reduced, rather than oxidised, fluids are liberated. Interestingly, if magnetite forms during seafloor hydration, the bulk rock Fe^3+^ content does not influence the excess oxygen-content of the fluids that evolve during subsequent dehydration since the system is buffered by the silicate + magnetite assemblage. Ocean floor-inherited Fe^3+^ content only becomes relevant after the chlorite dehydration reaction (3) when redox conditions are governed by equilibria involving ferric iron in clinopyroxene and garnet^36,41^. The reduced nature of aqueous fluids released from subducting ultramafic rocks affects the recycling capacity of redox-sensitive elements to arc magmas and the convecting mantle. The interaction of seawater with peridotites leads to an enrichment of U in serpentinites^42^. Recent findings on the trace element composition of subducted serpentinite have reported no mobilization of U during the brucite and antigorite dehydration reactions^43^, consistent with our results that indicate *f*O_2_ conditions close to QFM for reactions (1) and (2). Reducing conditions during chlorite dehydration (reaction 3) enable near complete retention of U beyond subarc depths in subducting, fully dehydrated, garnet metaperidotite. We therefore suggest that serpentinite-derived fluids cannot account for the observed elevated U/Th ratio in arc lavas^44–46^. Moreover, recent mass balance modelling has revealed that excess U relative to Th and Pb in ocean floor serpentinites recycled to the deep mantle offers a solution to the second terrestrial Pb-isotope paradox^47^, also known as the kappa conundrum^48^. Our findings support this hypothesis and emphasize the relevance of redox controls on element recycling in subduction zones.

The antigorite dehydration (reaction 2) generates fluids at fore-arc depth if serpentinites are located at the top of the slab. However, this reaction will occur at subarc depth in partially serpentinized peridotites within the cooler slab interior (Fig. 1). To date no information is available on the sulphide-oxide systematics of subducted oceanic mantle. Our study sets an upper bound on *f*O_2_ conditions for a potentially magnetite-free partially serpentinized peridotite as increasing pressure has a negligible effect on the position of the Fe-silicates - oxide mineral buffer.

Chlorite dehydration in serpentinites at the top of the slab occurs below the volcanic arc and may trigger partial melting of the mantle wedge (Fig. 1). Our model indicates that fluids produced upon the chlorite-breakdown reaction can be the source of S to sub-arc magmatism and may thus be relevant to the formation of magmatic-hydrothermal porphyry-type ore deposits. However, reduced conditions imply that S is present as H_2_S. Reduced mantle wedge conditions are indicated by studies on orogenic garnet peridotites that report subduction metasomatism with *f*O_2_ below the QFM buffer^49–51^. Aqueous fluids with reduced S species are in equilibrium with the mantle wedge mineral assemblage and thus could percolate until the magma source region.

Our new findings have important consequences for arc magma genesis. Four main processes have been proposed to explain why arc lavas are more oxidised than MORB: (1) transfer of oxidised species, especially S from the slab to the locus of partial melting in the mantle wedge^2,9,19,20^; (2) oxidation in the mantle during the ascent of hydrous magmas by dissociation of H_2_O in olivine and orthopyroxene^52,53^; (3) Fe^3+^ enrichment during differentiation of hydrous magmas at lower crustal conditions^54,55^; and (4) oxidation during degassing at the late stage of eruption/emplacement of magmas^40,56^. The results of this study indicate that the largely accepted view that oxidised slab fluids are the main cause for producing oxidised arc lavas has to be reconsidered. Our results could also explain why V/Sc and Zn/Fe, ratios used to determine the oxidation conditions of mantle partial melting, are indistinguishable in MORB and arc sources^40^.

The results we present in this work refer to slow/ultraslow spreading oceanic lithosphere. Nevertheless, the interaction of reduced serpentinite-derived fluids with thick altered oceanic crust and/or sediments that are present in fast-spreading lithosphere likely leads to the precipitation of sulphides, transformation of ferric to ferrous iron, and potentially to the formation of graphite/diamond during subsequent subduction. Thermodynamic calculations have shown that aqueous fluids equilibrated with altered oceanic crust are dominated by CO_2_^57^. However, we speculate that when such fluids are leaving the slab, interaction with a reduced garnet-peridotite mantle wedge might lead to a change in fluid speciation from CO_2_ to CH_4_ dominated aqueous fluids along with a concomitant oxidation of the mantle wedge only immediately above the slab^41^. Further studies are needed to better quantify the nature of these multiple interaction processes.

## Supplementary information

Supplementary Information
