# Supplementary material for: Subducting serpentinites release reduced, not oxidized, aqueous fluids
Source: Sci Rep. 2019 Dec 20;9:19573. doi: 10.1038/s41598-019-55944-8 (PMC6925189; doi:10.1038/s41598-019-55944-8)
Supplement: Supplementary file 1 — Supplementary Information [file 41598_2019_55944_MOESM1_ESM.docx]

**Subducting serpentinites release reduced, not oxidized, aqueous fluids**

Piccoli F.^1*^, Hermann J. ^1^, Pettke T. ^1^, Connolly J.A.D. ^2^, Kempf E. ^1^, Vieira Duarte J.F. ^1^

^1^University of Bern, Institute of Geological Sciences, Balzerstrasse 1+3, 3012 Bern, Switzerland

^2^Department of Earth Science, Swiss Federal Institute of Technology, Zurich, Switzerland

*corresponding author: francesca.piccoli@geo.unibe.ch

**Supplementary Information**

Phase diagram sections were calculated using the software Perple_X (version 6.8.6^1^) and the internally consistent thermodynamic database of Holland and Powell (2011)^2^ and the revision version ds6.22 combined with the Sverjensky et al. (2013) aqueous species data base for the Deep Earth Water (DEW)^3^ Model (DEW13HP622ver_oxide). Mineral solid solutions were used for olivine (O(HP))^4,5^, clinopyroxene (Cpx(HP)) ^4,5^, orthopyroxene (Opx(HP)) ^4,5^, chlorite (Chl(HP)) ^4,5^, garnet (Grt(HP))^4,5^, antigorite (Atg(PN))^6^, brucite (B), talc (T), tremolite (Tr), spinel (Sp(JH))^7^, pyrrhotite (Po(HP) ^4,5^. Hematite and iron are considered as pure phases. Fluids were modelled with the generic hybrid equation of state and the COH-Fluid+ solution model^8^. Phase diagram sections are calculated for the system Cr_2_O_3_-CaO-FeO-MgO-Al_2_O_3_-SiO_2_-H_2_O-S_2_ (SiO_2_ 37.12; Al_2_O_3_ 1.89; Cr_2_O_3_ 0.51; FeO 11.93; MgO 35.53; CaO 0.01; H_2_O 11.5; S_2_ 0.05-0.2), using a representative harzburgite bulk composition from Li et al. (2004) and assuming S_2_ content of 0.05 and 0.2 wt.%^10^. This composition is also consistent with published data of natural harzburgite from Cerro del Almirez^11^ and Cima di Gagnone^12^.

**Calculated oxygen fugacity**

In the ultramafic system, silicate-oxide equilibria are redox sensitive and thus indicate oxygen fugacity. This equilibrium is expressed by the reaction Fayalite + O_2_ = Magnetite + Quartz (Eq. 4, where the activity of silica in quartz is used as a proxy for the silica chemical potential which is buffered by coexisting Fe-Mg-silicates). With increasing *P* and T, the fayalite component in olivine decreases and the mineral-buffered silica activity increases, driving oxygen fugacity towards higher values. This can be plotted in a *p/T*-*f*O_2_ phase diagram section where the observed assemblage in Zermatt, Cerro del Almirez and Cima di Gagnone is reproduced at different *PT-f*O_2_ conditions (yellow stars in Fig. S1). We calculated the *PT*-*f*O_2_ phase diagram in Figure S1 using a geothermal gradient of 15 °C/km. By doing this, we assume that the mineral assemblage recorded the equilibrium with the fluid at specific P-T conditions along a prograde *P-T* path. The green arrow in Figure S1 indicates the *f*O_2_ evolution that can correctly predict the silicate-oxide assemblage observed in the natural samples and the calculated assemblages along the prograde *PT* path. This *PT*- *f*O_2_ path is the one reported in Figure 3, where *f*O_2_ values are given with a delta log notation to eliminate the *P-T* dependence of the absolute oxygen fugacity. Moreover, it also emphasises that in the QFM equilibrium (Eq. 4) both the olivine composition (affecting the activity of fayalite) as well as the Si-activity (as no free quartz is present in ultramafic rocks) will affect *f*O_2_. Olivine bearing serpentinite and olivine-orthopyroxene metaperidotite equilibrate at very different *f*O_2_ even if, in both cases, the stable oxide is magnetite. The reason is that the change in silicate assemblage (Br + Atg to Ol + Atg and Atg + Ol to Opx + Ol, respectively for reaction 1 and 2) progressively increases the silica activity from very low values (10^-2.3^) to higher values (10^-0.75^), while the olivine composition is moving towards more forsteritic compositions. This is shown for reaction 1 and 2 in Figures S2 and S3, respectively.

**Calculated fluid speciation**

Measured bulk Fe^3+^ content might be subject to large biases related to the presence of retrograde phases such as hematite. In order to avoid this bias, we used the bulk composition of the stability field of Atg+Br+Chl+Cpx+Chl+Mt+Po (orange symbol in Fig. S1; composition obtained by meemum routine in Perple_X) and fix the initial Fe^3+^/Fe^2+^ ratio by adding oxygen as a component (0_2_=0.348 wt.%). We use this bulk as input value for a *P-T* phase diagram calculated with the thermodynamic data file DEW13HP622ver_oxides (Fig. S4). From this phase diagram, we calculated the total S-content of the fluid using both a simple molecular model, with no dissolved solutes, and electrolyte fluid model (Table S1). In the latter case, the solute load was computed by simple back-calculation^8^. The H_2_S molar proportion of the fluid for the molecular fluid model is shown in Figure S4 and H_2_S values for reaction 1, 2, 3 are also reported.

**Fig. S1:** p/T vs. *f*O_2_ section along a linear geothermal gradient of 15 °C/km. The green line represents the *fO_2_* evolution deduced from silicate-oxide assemblage. Modelled bulk with S content of 0.2 wt.%. Phase diagram section for a lower S content of 0.05 wt% gives identical results. Solid buffers (dashed lines) were calculated by *PT*-*f*O_2_ Schreinemaker diagrams in FeO-SiO_2_ and FeO-Al_2_O_3_-MgO-SiO_2_ systems to account for silicate XMg number (i.e. Mg/Mg+Fe).

**Fig. S2:** *T* vs. aSiO_2_ (i.e. activity of quartz, aSiO_2_) isobaric section. Field of brucite + antigorite + olivine + chlorite is in yellow, antigorite + olivine + chlorite + fluid in violet. Isopleths for olivine composition (XFo: fosterite /fosterite+fayalite) are also reported. The blue arrow indicates that the crystallization of olivine drives an increase in SiO_2_ activity of ca. 1 log unit. Red dashed line indicates the threshold in aSiO_2_ above which brucite is no longer stable. Modelled pressure is 2.5 GPa corresponding to the pressure peak of the Zermatt-Saas unit


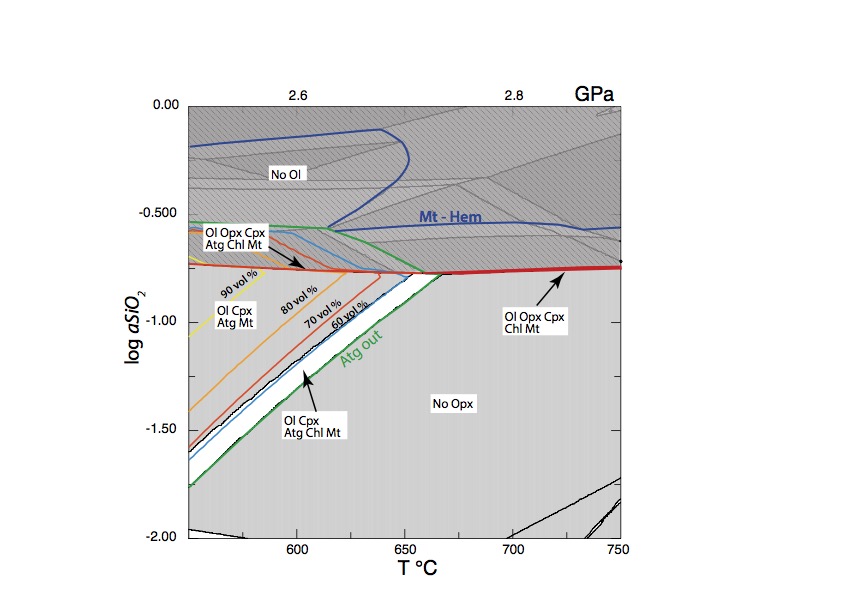


**Fig. S3:** *PT-*SiO_2_ activity phase diagram section showing that the assemblage Atg+Ol+Chl+Mt with increasing T equilibrates at higher Si activity. When Atg is totally consumed, Si activity is buffered by Opx+Ol (red line). Shaded area above the red line indicates fields where olivine is not stable. Blue line indicates the magnetite-hematite transition. Coloured isopleths for antigorite modes (volume abundance) are also reported.

**Fig. S4:** P-T section calculated from the bulk composition in Fig. S1 at 2 GPa, 450°C and *fO_2_* = 10^-27^ corresponding to the antigorite + brucite + chlorite + magnetite + pyrrhotite field (orange symbol in Fig. S1). Fluid H_2_S molar proportions for the molecular fluid models are depicted with colour code (minimum value, blue colour, 0; maximum value, red colour, 1.6 10^-3^). H_2_S values for P-T points of each dehydration reaction are also reported.

REFERENCES:

1. Connolly, J. The geodynamic equation of state: what and how. *Geochemistry, Geophysics, Geosystems* **10**, (2009).

2. Holland, T. & Powell, R. An improved and extended internally consistent thermodynamic dataset for phases of petrological interest, involving a new equation of state for solids. *Journal of Metamorphic Geology* **29**, 333–383 (2011).

3. Sverjensky, D. A., Harrison, B. & Azzolini, D. Water in the deep Earth: the dielectric constant and the solubilities of quartz and corundum to 60 kb and 1200 C. *Geochimica et Cosmochimica Acta* **129**, 125–145 (2014).

4. Holland, T. J. B. & Powell, R. An internally consistent thermodynamic data set for phases of petrological interest. *Journal of Metamorphic Geology* **16**, 309–343 (1998).

5. Holland, T. & Powell, R. Thermodynamics of order-disorder in minerals: II. Symmetric formalism applied to solid solutions. *American Mineralogist* **81**, 1425–1437 (1996).

6. Padrón-Navarta, J. A. *et al.* Tschermak’s substitution in antigorite and consequences for phase relations and water liberation in high-grade serpentinites. *Lithos* **178**, 186–196 (2013).

7. Jennings, E. S. & Holland, T. J. A simple thermodynamic model for melting of peridotite in the system NCFMASOCr. *Journal of Petrology* **56**, 869–892 (2015).

8. Connolly, J. A. & Galvez, M. E. Electrolytic fluid speciation by Gibbs energy minimization and implications for subduction zone mass transfer. *Earth and Planetary Science Letters* **501**, 90–102 (2018).

9. Li, X., Rahn, M. & Bucher, K. Serpentinites of the Zermatt‐Saas ophiolite complex and their texture evolution. *Journal of Metamorphic Geology* **22**, 159–177 (2004).

10. Alt, J. C. *et al.* The role of serpentinites in cycling of carbon and sulfur: seafloor serpentinization and subduction metamorphism. *Lithos* **178**, 40–54 (2013).

11. Padrón-Navarta, J. A., Lopez Sanchez-Vizcaino, V., Garrido, C. J. & Gómez-Pugnaire, M. T. Metamorphic record of high-pressure dehydration of antigorite serpentinite to chlorite harzburgite in a subduction setting (Cerro del Almirez, Nevado–Filábride Complex, Southern Spain). *Journal of Petrology* **52**, 2047–2078 (2011).

12. Pfiffner, M. A. Genese der hochdruckmetamorphen ozeanischen Abfolge der Cima Lunga-Einheit (Zentralalpen). (1999).
